# Optimizing plant size for vertical farming by editing stem length regulators

**DOI:** 10.1111/pbi.70129

**Published:** 2025-05-09

**Authors:** Yoonseo Lim, Myeong‐Gyun Seo, Jiwoo Lee, Seungpyo Hong, Jeong‐Tak An, Ho‐Young Jeong, Hong‐Il Choi, Woo‐Jong Hong, Chanhui Lee, Soon Ju Park, Choon‐Tak Kwon

**Affiliations:** ^1^ Graduate School of Green‐Bio Science Kyung Hee University Yongin Korea; ^2^ Department of Smart Farm Science Kyung Hee University Yongin Korea; ^3^ Department of Plant & Environmental New Resources Kyung Hee University Yongin Korea; ^4^ Advanced Radiation Technology Institute Korea Atomic Energy Research Institute Jeongeup Korea; ^5^ Division of Applied Life Science, Plant Molecular Biology and Biotechnology Research Center Gyeongsang National University Jinju South Korea; ^6^ Present address: Department of Seed R&D ToolGen Inc. Cheongju Korea

**Keywords:** vertical farming, CRISPR, tomato, gibberellin, GA3ox, plant size

## Abstract

Vertical farming offers the advantage of providing a stable environment for plant cultivation, shielding them from adverse conditions such as climate change. For fruit‐harvesting plants like tomato, vertical farming necessitates the optimization of plant growth and architecture. The *gibberellin 3‐oxidase* (*GA3ox*) genes encode gibberellin 3‐oxidases responsible for activating GA within the pathway and modulating stem length. Among the five *SlGA3ox* genes, we targeted the coding regions of three *SlGA3ox* genes (named *SlGA3ox3*, *SlGA3ox4* and *SlGA3ox5*) using multiplex CRISPR genome editing. The *slga3ox4* single mutants exhibited a slight reduction in primary shoot length, leading to a smaller stature. In contrast, the *slga3ox3* and *slga3ox5* single mutants showed subtle phenotypic changes. Notably, the *slga3ox3 slga3ox4* double mutants developed a more compact shoot architecture with minor physiological differences, potentially making them suitable for vertical farming applications. We observed a correlation between total yield and plant size across all genotypes through multiple yield trials. Observations from vertical farm cultivation revealed that *slga3ox3 slga3ox4* plants possess a markedly compact plant size, offering potential benefits for space‐efficient cultivation. Our research suggests that targeted manipulation of hormone biosynthetic genes can effectively tailor plant architecture for vertical farming.

## Introduction

Currently, the rapid increase in natural disasters due to drastic climate change is impacting agriculture in profound ways (Benevolenza and DeRigne, 2019). In response to worsening environmental conditions, new technologies such as Information and Communication Technology (ICT) and the Internet of Things (IoT) are being applied to agriculture (El‐Beltagy and Madkour, 2012). These advanced technologies allow remote and automatic control of growing environments and real‐time collection of environmental data (Kim *et al*., 2020; Pallavi *et al*., 2017). ICT and IoT have also furthered agricultural methods such as hydroponics and vertical farming (Benke and Tomkins, 2017; Wollschlaeger *et al*., 2017). Vertical farming aims to boost production in constrained spaces by stacking crops vertically, effectively increasing the yield per area (Benke and Tomkins, 2017; van Delden *et al*., 2021). This method is particularly effective for leafy greens, which are well suited to compact spaces and can be harvested rapidly once they mature (Kwon, 2023). In contrast, fruit crops, which generally have larger sizes or slower growth cycles, are less suited to vertical farming environments (Kwon, 2023; van Delden *et al*., 2021). While leafy greens are commercially valued for their total biomass, fruit crops are primarily marketed based on their harvested fruits. Therefore, there is a need for corresponding changes in the crops being cultivated alongside the advancement of agricultural systems (Beacham *et al*., 2019).

To optimize fruit crops for urban agriculture and vertical farming with early flowering and compact architecture, several genes have been modified through random mutagenesis and genome editing. For example, in *Solanum lycopersicum* (tomato), The *SELF PRUNING* (*SP*) gene critically shapes shoot architecture, where *SP* mutations lead to earlier shoot termination (Pnueli *et al*., 1998). This reduces overall shoot size and levels out growth across inflorescences, ensuring uniform fruit maturation and facilitating efficient mechanical harvest to maximize productivity (Pnueli *et al*., 1998). *SELF PRUNING 5G* (*SP5G*), a paralog of *SP*, delays flowering (Zhang *et al*., 2018a). Mutations in *SP5G* result in fewer leaves before the first inflorescence, thus accelerating fruit production (Soyk *et al*., 2017). The *sp sp5g* double mutants, created through CRISPR‐Cas9 and known as ‘double‐determinate’ combine the benefits of determinate growth and early flowering with reduced plant size (Soyk *et al*., 2017). Further modification of the stem internode length regulator, *SlERECTA* (*SlER*), has produced ‘triple‐determinate’ *sp sp5g sler* plants, which are well‐suited for confined spaces in urban farming (Kwon *et al*., 2020). However, it remains to be confirmed whether triple‐determinate plants can effectively thrive in indoor vertical farming (Kwon, 2023).

Efforts to maximize productivity by tailoring plant size to specific growing conditions have evolved from traditional breeding to modern techniques. Historically, modifications in plant stature have been achieved by manipulating the signalling pathways or biosynthesis of hormones such as gibberellin (GA), brassinosteroid (BR), strigolactone (SL) and auxin (Busov *et al*., 2008). Notably, *semidwarf1* (*sd‐1*) and *Reduced height* (*Rht*), pivotal in the Green Revolution, have been crucial in modifying plant height to boost *Oryza sativa* (rice) and *Triticum aestivum* (wheat) yields by influencing GA biosynthesis and signalling (Hedden, 2003). Additionally, natural variations in *Flowering Promoting Factor 1* (*FPF1*), linked to GA signalling, have been leveraged in rice and tomato to cultivate shorter stem internodes for crop domestication (Lee *et al*., 2022; Nagai *et al*., 2020). Recent studies have also revealed the impact of BR on crop size, with notable insights from the miniature tomato ‘Micro‐Tom’, identifying a correlation between BR and dwarfism (Martí *et al*., 2006; Song *et al*., 2023). Particularly, mutations in the *D* gene, which encodes cytochrome P450 for BR biosynthesis, have been shown to decrease stem length and overall shoot size in tomato plants (Bishop *et al*., 1999). Additionally, modifications in the biosynthesis or signalling of SL and auxin have similarly been documented to affect stem length and plant size, demonstrating the broad applicability of hormonal control in plant architecture optimization (Ma *et al*., 2016; Zhang *et al*., 2018b).

Among various plant hormones, GA plays an essential role in various growth processes, including seed germination, leaf expansion, flowering and fruit set and is key in regulating stem length and overall plant size (Chen *et al*., 2016; Min *et al*., 2022). GA20‐oxidase (GA20ox) and GA3‐oxidase (GA3ox) are the primary enzymes responsible for synthesizing the active forms of GA, triggering subsequent signalling cascades (Rieu *et al*., 2008). Specifically, bioactive GAs interact with a receptor, GIBBERELLIN‐INSENSITIVE DWARF1 (GID1), promoting the degradation of DELLA proteins and thus initiating GA signalling (Sun, 2011). *Arabidopsis thaliana* (thale cress; hereafter *Arabidopsis*) and tomato each harbour three functional *GID1* orthologues, while rice features a single *GID1* gene (Gazara *et al*., 2018). DELLA proteins, named for five conserved amino acids in their N‐terminal domain, primarily inhibit GA responses (Phokas and Coates, 2021). *Arabidopsis* expresses five DELLA: GIBBERELLIC ACID INSENSITIVE (GAI), REPRESSOR‐OF‐ga1‐3 (RGA), RGA‐LIKE1 (RGL1), RGL2 and RGL3, with RGA and GAI mainly controlling stem elongation (Cheng *et al*., 2004). Unlike *Arabidopsis*, which has multiple DELLA genes, rice, barley and tomato each have a single DELLA: SLENDER RICE1 (SLR1), SLENDER1 (SLN1) and PROCERA (PRO), respectively (Phokas and Coates, 2021). Genetic disruptions in *GA20ox* and *GA3ox* reduce GA levels, leading to DELLA protein accumulation, restricting stem elongation and inducing dwarfism (Mitchum *et al*., 2006; Zhang *et al*., 2020). Such dwarfism, prompted by alterations in *GA20ox* and *GA3ox*, is consistently observed across various crops, including *Solanum tuberosum* (potato), *Citrullus lanatus* (watermelon), *Cucurbita Moschata* (pumpkin), *Pisum sativum* (pea) and *Cucumis sativus* (cucumber) (Zhang *et al*., 2022).

Here, we aimed to optimize plant size and structure through CRISPR genome editing to enhance the suitability of fruit crops for vertical farming environments. Our study focused on the tomato, specifically the ‘Sweet100’ cherry tomato cultivar, recognized for its rapid maturation from sowing to fruiting and high seed count per fruit (Alonge *et al*., 2022). Additionally, its lower nutrient requirements for fruit maturation and the manageability of its small fruit size facilitate the maintenance of plants form post‐ripening, compared to larger fruit varieties (Kwon *et al*., 2020; O'Sullivan *et al*., 2019). Our initial development of Sweet100 *sp sp5g sler* (triple‐determinate) plants revealed that their stem length and plant size were not optimal for vertical farming, leading us to undertake additional genetic modifications to reduce the stem length (Figure S1). Subsequent phenotypic characterization and yield trials demonstrated that the genetically modified plants exhibit an architecture well‐suited for vertical farming. Our study supports the potential of precise genetic modifications to develop specialized fruit varieties for vertical farming, paving the way for future innovations in agricultural practices.

## Results

### Identification of five tomato *SlGA3ox* genes

**Figure 1:**
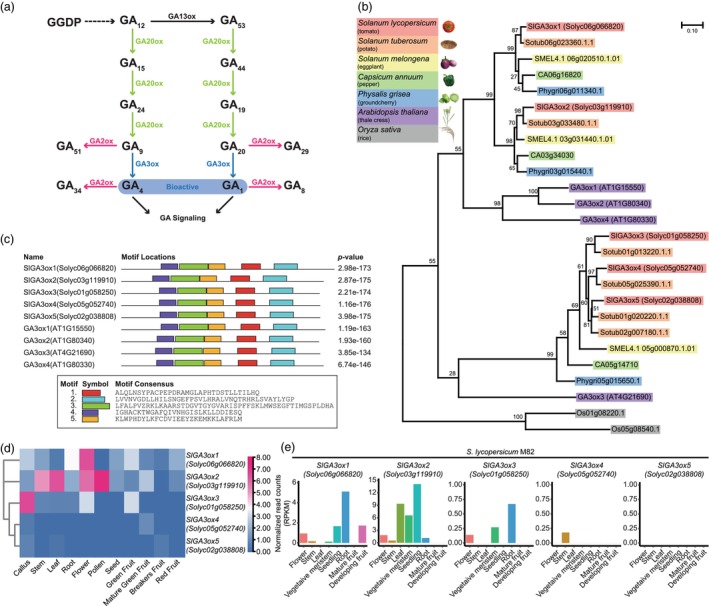
Identification of five tomato *SlGA3ox* genes. (a) The pathway of GA biosynthesis in plants. Biologically active GAs are emphasized within a blue box. GGDP, geranylgeranyl diphosphate; (b) Phylogenetic tree of the *GA3ox* gene family in tomato, potato, eggplant, pepper, groundcherry, *Arabidopsis* and rice. Bootstrap values from 1000 replicates are represented on each node. (c) Motif composition of *GA3ox* genes and *SlGA3ox* genes using MEME. (d) Heatmap of transcript abundance of *SlGA3ox* genes in different tissues using CRISPR applicable functional redundancy inspector (CAFRI)‐Tomato. The colour of the cell represents transcript abundance calculated by normalized RPKM. Blue‐coloured boxes and red‐coloured boxes denote a low level of expression and a high level of expression, respectively. (e) Normalized read counts for five *SlGA3ox* genes in multiple tissues from Rose Lab Atlas in the Tomato eFP Browser.

GA3ox is the final enzyme involved in the synthesis of bioactive GAs, and we hypothesized that editing tomato orthologs of *GA3ox* genes could optimize stem internode length for vertical farming (Figure 1a). To develop a new tomato cultivar suitable for vertical farming, we first identified the *SlGA3ox* genes within the tomato genome and ultimately discovered five distinct *SlGA3ox* genes from phylogenetic analysis (Figure 1b,c). Next, we investigated the evolutionary conservation of *SlGA3ox* genes from the phylogenetic tree of tomato, potato, *Solanum melongena* (eggplant), *Capsicum annuum* (pepper) and *Physalis grisea* (groundcherry), *Arabidopsis* and rice (Figure 1b). The phylogenetic analysis revealed that the *SlGA3ox* genes are conserved across the Solanaceae species analysed (Figure 1b). Notably, *SlGA3ox1* and *SlGA3ox2*, along with *Arabidopsis GA3ox1*, *GA3ox2* and *GA3ox4*, are grouped together, while *SlGA3ox3*, *SlGA3ox4* and *SlGA3ox5* are conserved along with *Arabidopsis GA3ox3* (Figure 1b). Additionally, *SlGA3ox3*, *SlGA3ox4* and *SlGA3ox5* show similar gene structures, suggesting that the three *SlGA3ox*s are the closest paralogs (Figure 1c; Figure S2). Using Multiple Expectation Maximization for Motif Elicitation (MEME) analysis, we found that the SlGA3ox protein sequences display motif locations similar to those in *Arabidopsis* GA3ox protein sequences (Figure 1c; Figure S2a).

The expression patterns of five *SlGA3ox* genes were analysed using tomato gene expression databases. We observed that the expression of *SlGA3ox3*, *SlGA3ox4* and *SlGA3ox5* was relatively lower compared to the other two *SlGA3ox* genes (Figure 1d,e; Figure S3a). Notably, *SlGA3ox4* showed specific expression in the stem, though at lower levels (Figure 1e). Since our goal was to develop a new cultivar based on the triple‐determinate background, we further examined the tissue‐specific expression patterns of *SlGA3ox* genes in triple‐determinate plants. The observed expression profiles were largely consistent with patterns reported in public expression databases (Figure S3b). The varied expression among these genes suggests tissue‐specific or functional distinctions within the plant, prompting us to target multiple redundant genes to fine‐tune plant size. Moreover, active sites of GA action and accumulation may not always align with transcriptional expression patterns alone (Binenbaum *et al*., 2018). Since GA biosynthesis and GA signalling do not always occur in the same tissues, targeting SlGA3ox genes with relatively low expression in the stem may still result in substantial phenotypic alterations. We thus selected *SlGA3ox3*, *SlGA3ox4* and *SlGA3ox5*, instead of *SlGA3ox1* and *SlGA3ox2*, to modulate stem length due to their relatively low expression levels and close phylogenetic relationship as paralogs.

### Shoot architecture of *slga3ox3*, *slga3ox4* and *slga3ox5* plants under hydroponic conditions

**Figure 2:**
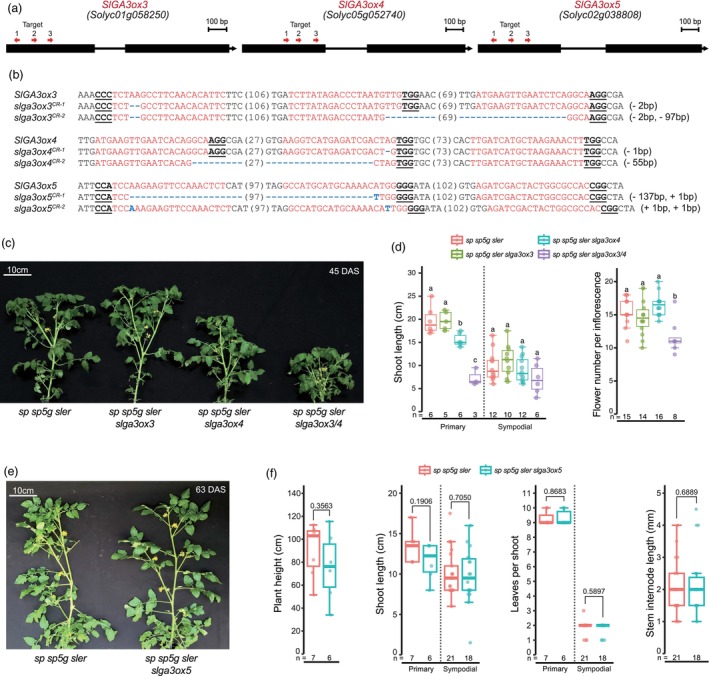
CRISPR targeted mutagenesis of *SlGA3ox* genes. (a) Gene structures and guide RNA information of *SlGA3ox3*, *SlGA3ox4* and *SlGA3ox5*. Black boxes indicate exon. Red arrows indicate the guide RNAs. (b) Sequences of *SlGA3ox3*, *SlGA3ox4* and *SlGA3ox5* mutant alleles generated by CRISPR–Cas9. Guide RNA and protospacer‐adjacent motif (PAM) sequences are highlighted in red and bold underlined, respectively. Blue dash and letter indicate deletion and insertion. Numbers in parentheses indicate gap lengths. (c) Shoots of *sp sp5g sler*, *sp sp5g sler slga3ox3*, *sp sp5g sler slga3ox4* and *sp sp5g sler slga3ox3/4* in the ebb and flow beds. DAS, days after sowing; (d) Shoot length and flower number per inflorescence of *sp sp5g sler*, *sp sp5g sler slga3ox3*, *sp sp5g sler slga3ox4* and *sp sp5g sler slga3ox3/4* in the ebb and flow beds. Different letters between genotypes indicate significance. Primary shoots, between 1st inflorescence and 1st leaf of the primary shoot; Sympodial shoot, between 1st and 2nd inflorescences and between 2nd and 3rd inflorescences; Flower number per inflorescence, number of flowers from 1st–3rd inflorescence; (e) Shoots of *sp sp5g sler* and *sp sp5g sler slga3ox5* in the ebb and flow beds. (f) Plant height, shoot length, leaves per shoot and stem internode length from *sp sp5g sler* and *sp sp5g sler slga3ox5* in the ebb and flow beds. Statistical analyses were performed using a two‐tailed, two‐sample *t*‐test. Plant height, length between last inflorescence and 1st leaf; Leaves per primary shoot, number of leaves from 1st leaf to 1st inflorescence; Leaves per sympodial shoot, number of leaves between 1st–2nd, 2nd–3rd and 3rd–4th inflorescences; Stem internode, internode between the 5th–6th, 6th–7th and 7th–8th leaves of the primary shoot; Number of inflorescence, number of inflorescence in the main shoot; (d and f) Box plots, 25th–75th percentile; center line, median; whiskers, full data range. Exact sample sizes (*n*) for replicate types are indicated. Letters indicate significance groups at *P* < 0.05 (one‐way ANOVA and Tukey HSD). At least two experiments were repeated independently with similar results.

We utilized multiplex CRISPR targeting on *SlGA3ox3*, *SlGA3ox4* and *SlGA3ox5* of triple‐determinate plants, applying three guide RNAs for each gene (Figure 2a). Among three *SlGA3ox* genes, *SlGA3ox3* and *SlGA3ox4* were simultaneously targeted due to their relatively high expression levels, leading to the generation of transgene‐free *slga3ox3 slga3ox4* homozygous plants (*slga3ox3/4*) from second‐generation transgenic (T_1_) plants. Additionally, transgene‐free *slga3ox3* and *slga3ox4* single mutant plants were obtained from second filial (F_2_) generation using backcrossing with triple‐determinate plants. This approach resulted in multiple alleles for each targeted gene, leading to frameshift mutations and premature stop codons (Figure 2b; Figure S4). To characterize phenotype of CRISPR‐generated lines, we selected transgene‐free homozygous mutant plants and cultivated the plants in an in‐house developed automated ‘ebb and flow’ hydroponic system across two trials (Lim *et al*., 2024) (Figure S5a). We first confirmed that there were no phenotypic differences among the allelic variants of each gene and subsequently used the *slga3ox3* ^*CR‐1*^ single, *slga3ox4* ^*CR‐2*^ single and *slga3ox3* ^*CR‐2*^ *slga3ox4* ^*CR*‐1^ double mutants for further phenotypic analysis. We found subtle phenotypic differences in primary and sympodial shoot length of *slga3ox3* and *slga3ox4* single mutants compared to triple‐determinate plants, though *slga3ox4* single mutants showed a slight reduction of primary shoot length (Figure 2c,d; Figure S5b,c). However, the *slga3ox3/4* double mutants exhibited a significant reduction in primary shoot length, more than 50% shorter than that of triple‐determinate plants (Figure 2c,d; Figure S5b,c). This dwarfism in the *slga3ox3 slga3ox4* double homozygous mutants was consistently observed across four CRISPR‐generated lines derived from independent first‐generation transgenic (T_0_) plants (Figure S6a,b). Additionally, we observed that *slga3ox3*/*4* plants exhibited a lower overall germination rate and delayed germination timing compared to triple‐determinate plants (Figure S6c).

Next, we examined additional phenotypic changes such as the number of leaves to the first inflorescence and the number of flowers per inflorescence. We found that no significant differences in the number of leaves to the first inflorescence were observed among the four genotypes, although the number was slightly lower in *slga3ox3/4* double mutants in the second trial (Figure S5b,c). However, the number of flowers per inflorescence was significantly reduced in the *slga3ox3/4* double mutants (Figure 2d; Figure S5c). Additionally, we observed that inflorescence stem length showed a similar trend to the number of flowers per inflorescence, particularly in *slga3ox3/4* double mutants, which had shorter overall inflorescence stem lengths compared to triple‐determinate plants (Figure S5b,c). In transgene‐free *slga3ox5* single mutants, no changes were found in primary and sympodial shoot lengths or in the number of leaves per primary and sympodial shoots (Figure 2e,f).

### Shoot architecture of *slga3ox3*, *slga3ox4* and *slga3ox3/4* plants in cocopeat cultivation

**Figure 3:**
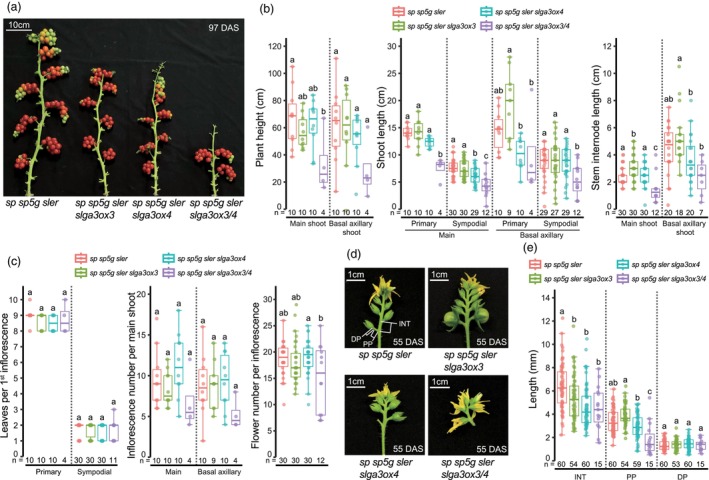
Shoot and inflorescence architecture of *slga3ox3*, *slga3ox4* and *slga3ox3/4* mutants. (a) Shoots of *sp sp5g sler*, *sp sp5g sler slga3ox3*, *sp sp5g sler slga3ox4* and *sp sp5g sler slga3ox3/4*. (b) Plant height, shoot length and stem internode length of *sp sp5g sler*, *sp sp5g sler slga3ox3*, *sp sp5g sler slga3ox4* and *sp sp5g sler slga3ox3/4*. Plant height, length between last inflorescence and 1st leaf; Primary shoots, between 1st inflorescence and 1st leaf of the primary shoot; Sympodial shoot, between 1st–2nd, 2nd–3rd and 3rd–4th inflorescences; Stem internode of main shoot, internode between the 5th–6th, 6th–7th and 7th–8th leaves of the primary shoot; Stem internode of basal axillary shoot, internode between 4th–5th and 5th–6th leaves of primary shoot; (c) Leaves per shoot, inflorescence number per main shoot, and number of flowers per inflorescence of *sp sp5g sler*, *sp sp5g sler slga3ox3*, *sp sp5g sler slga3ox4* and *sp sp5g sler slga3ox3/4*. (d) 2nd inflorescence of *sp sp5g sler*, *sp sp5g sler slga3ox3*, *sp sp5g sler slga3ox4* and *sp sp5g sler slga3ox3/4*. (e) INT, PP and DP length of *sp sp5g sler*, *sp sp5g sler slga3ox3*, *sp sp5g sler slga3ox4* and *sp sp5g sler slga3ox3/4*. INT, inflorescence internode between 1st–2nd, 2nd–3rd and 3rd–4th flowers of 2nd inflorescence; PP, proximal section of 1st–3rd pedicels; DP, distal section of 1st–3rd pedicels. (a–e) The plants grown from September 19, 2023 to January 09, 2024. (b, c and e) Box plots, 25th–75th percentile; center line, median; whiskers, full data range. Exact sample sizes (*n*) for replicate types are indicated. Letters indicate significance groups at *P* < 0.05 (one‐way ANOVA and Tukey HSD). Different letters between genotypes indicate significance. At least two experiments were repeated independently with similar results.

We conducted two rounds of growth trials using cocopeat to verify the phenotypic consistency of shoot and inflorescence architecture, previously observed in the ebb and flow hydroponic system (Figure S7a). The phenotypes of the *slga3ox3*, *slga3ox4* and *slga3ox3/4* mutant plants, particularly related to shoot architecture, showed consistent phenotypic output along with minimal variation across hydroponics and cocopeat trials. We noted some variations in the shoot length of the *slga3ox3/4* double mutants between both cocopeat trials (Figure 3a,b; Figure S7b,c). Nevertheless, *slga3ox3/4* double mutants showed significantly shortened stem internode lengths, reducing the total length (plant height) of the main and basal axillary shoots to more than 50 % shorter than that of triple‐determinate plants (Figure 3a,b). Contrary to the *slga3ox3/4* double mutants, we consistently observed minimal phenotypic changes in the *slga3ox3* and *slga3ox4* single mutants across both trials (Figure 3a,b; Figure S7b,c).

To explore whether the shoot architecture of the *slga3ox3/4* double mutants derived solely from changes in stem internode length, we measured the number of leaves per primary and sympodial shoots and found no differences between the four genotypes in either trial (Figure 3c; Figure S7d). However, the *slga3ox3/4* double mutants showed a slight reduction in the number of inflorescences per main shoot and a significant reduction in flowers per inflorescence, which correlated with their reduced plant stature (Figure 3c; Figure S7d). The effect of the mutations on stem internode length was mirrored in the inflorescences, showing similar trends (Figure 3d,e; Figure S7e,f). These results confirm that the shoot architecture of *slga3ox3*, *slga3ox4* and *slga3ox3/4* mutant plants is consistent across both hydroponic and cocopeat media, underscoring the potential of the highly compact stature of the *slga3ox3/4* double mutants for applications in vertical farming.

### Physiological analyses of *slga3ox3*, *slga3ox4* and *slga3ox3/4* plants

**Figure 4:**
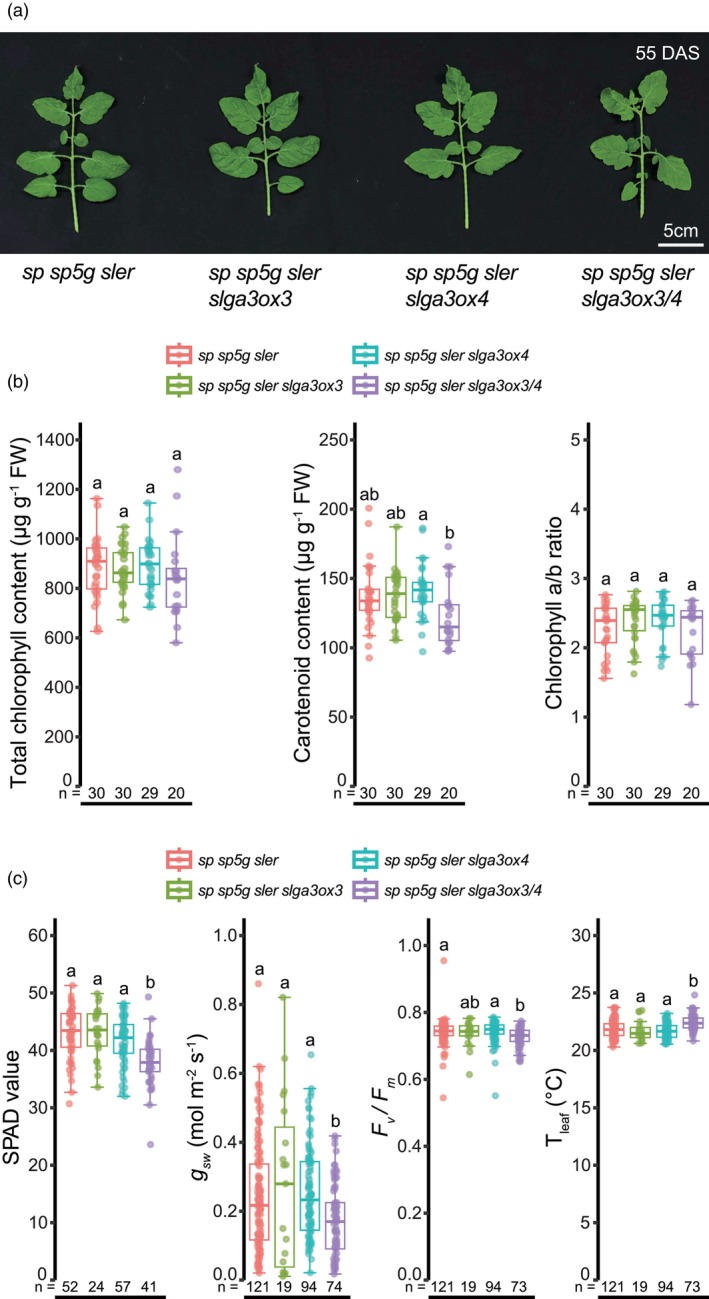
Physiological analysis of *slga3ox3*, *slga3ox4* and *slga3ox3/4* mutants. (a) 7th leaf of *sp sp5g sler*, *sp sp5g sler slga3ox3*, *sp sp5g sler slga3ox4* and *sp sp5g sler slga3ox3/4*. (b) Total chlorophyll content, carotenoid content and chlorophyll a/b ratio of *sp sp5g sler*, *sp sp5g sler slga3ox3*, *sp sp5g sler slga3ox4* and *sp sp5g sler slga3ox3/4*. (c) SPAD value, g_sw_, *F*_*v*_ */F*_*m*_ and T_leaf_ of *sp sp5g sler*, *sp sp5g sler slga3ox3*, *sp sp5g sler slga3ox4* and *sp sp5g sler slga3ox3/4*. (a–c) The plants grew from September 19, 2023 to January 09, 2024. (b and c) Box plots, 25th–75th percentile; center line, median; whiskers, full data range. Exact sample sizes (*n*) for replicate types are indicated. Letters indicate significance groups at *P* < 0.05 (one‐way ANOVA and Tukey HSD). Different letters between genotypes indicate significance. At least two experiments were repeated independently with similar results.

Next, we assessed physiological analyses of the leaves aside from alterations in shoot architecture due to the *slga3ox3* and *slga3ox4* mutations (Figure 4; Figure S8). We found that the leaf colour of *slga3ox3*, *slga3ox4* and *slga3ox3/4* mutant plants was indistinguishable, though some leaves of the *slga3ox3/4* mutants exhibited slight variations (Figure 4a; Figure S8a). We therefore examined total chlorophyll and carotenoid content and found no significant differences in pigment levels, although *slga3ox3/4* mutants showed a slight decrease in both chlorophyll and carotenoid content in some trials (Figure 4b; Figure S8b,d). Additionally, the chlorophyll a/b ratio showed minor variation among the four genotypes (Figure 4b; Figure S8b,d,f).

To investigate whether the mutations affect photosynthetic pigment formation and other physiological traits in more detail, we evaluated the Soil Plant Analysis Development (SPAD) value, stomatal conductance (g_sw_) and photosynthetic efficiency (*F*_*v*_/*F*_*m*_) using non‐destructive phenotypic analyses (Figure 4c; Figure S8b,c,e). Interestingly, we found that the *slga3ox3/4* double mutants exhibited similar or slightly lower SPAD, g_sw_ and *F*_*v*_/*F*_*m*_ values compared to the other genotypes in both ebb and flow hydroponics and cocopeat cultivation conditions, indicating that the double mutations in *SlGA3ox3* and *SlGA3ox4* affect chlorophyll content and photosynthetic efficiency (Figure 4c; Figure S8b,c,e). These results suggest that the subtle decrease in chlorophyll content might contribute to the reduction in photosynthetic efficiency observed in the *slga3ox3/4* double mutants.

Furthermore, stomatal conductance in the *slga3ox3/4* double mutants was significantly reduced compared to the other genotypes, suggesting that the lower frequency of stomatal opening in these mutants negatively affects CO_2_ absorption for photosynthesis (Figure 4c; Figure S8c,e). Additionally, the temperature of the leaf surface (T_leaf_) was slightly but significantly increased in the *slga3ox3/4* double mutants, opposite to the values observed for stomatal conductance (Figure 4c; Figure S8c,e). Overall, our data suggest that the altered pattern of stomatal opening results in variations in photosynthetic efficiency and leaf temperature.

### Yield and yield‐related traits of *slga3ox3*, *slga3ox4* and *slga3ox3/4* plants

**Figure 5:**
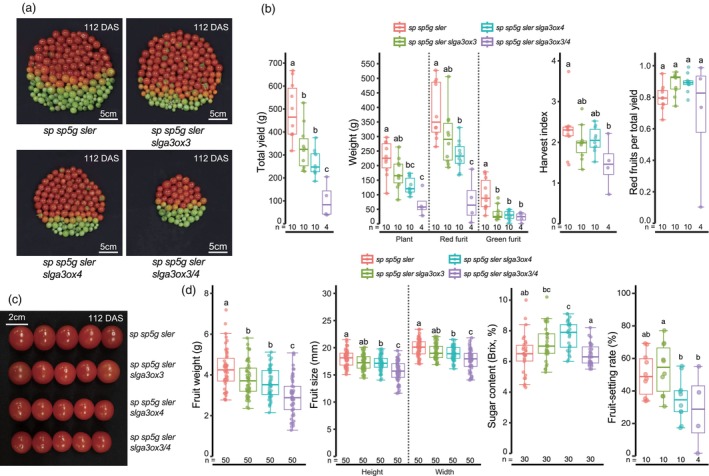
Yield and yield‐related traits of *slga3ox3*, *slga3ox4* and *slga3ox3/4* mutants. (a) Total fruits of *sp sp5g sler*, *sp sp5g sler slga3ox3*, *sp sp5g sler slga3ox4* and *sp sp5g sler slga3ox3/4*. (b) Total yield, weight, harvest index and red fruits per total yield of *sp sp5g sler*, *sp sp5g sler slga3ox3*, *sp sp5g sler slga3ox4* and *sp sp5g sler slga3ox3/4*. (c) The five fruits with the average width of *sp sp5g sler*, *sp sp5g sler slga3ox3*, *sp sp5g sler slga3ox4* and *sp sp5g sler slga3ox3/4*. (d) Fruit weight, fruit length, sugar content and fruit‐setting rate of *sp sp5g sler*, *sp sp5g sler slga3ox3*, *sp sp5g sler slga3ox4* and *sp sp5g sler slga3ox3/4*. (c and d) The plants grown from September 19, 2023 to January 09, 2024. (b and d) Box plots, 25th–75th percentile; center line, median; whiskers, full data range. Exact sample sizes (*n*) for replicate types are indicated. Letters indicate significance groups at *P* < 0.05 (one‐way ANOVA and Tukey HSD). Different letters between genotypes indicate significance. At least two experiments were repeated independently with similar results.

We investigated whether mutations in the *SlGA3ox3* and *SlGA3ox4* led to changes in fruit productivity and quality. First, we examined the total fruit yield, plant weight and harvest index (total yield/plant weight) of triple‐determinate, *slga3ox3*, *slga3ox4* and *slga3ox3/4* plants across two cocopeat cultivation trials (Figure 5; Figure S9). We found that total yield and plant weight were proportional to the size of each genotype (Figure 5b; Figure S9b). Specifically, the *slga3ox3/4* double mutants exhibited a significant reduction in total fruit yield per plant due to their compact size, with a corresponding slight decrease in harvest index (Figure 5b; Figure S9b). Additionally, the *slga3ox3/4* plants demonstrated notable deviations in the ratio of red fruit to total yield, generally resulting in lower values compared to the other genotypes (Figure 5b; Figure S9b). This reduction in yield per individual possibly correlates with decreases in inflorescence and flower numbers (Figure 3c; Figures S6c and S7d).

To further investigate yield differences, we analysed individual fruit weight and size. Across two independent yield trials, *slga3ox3* and *slga3ox4* single mutants exhibited only minor reductions in fruit weight, whereas *slga3ox3*/*4* double mutants showed a significant decrease compared to triple‐determinate plants (Figure 5c,d; Figure S9c,d). The reduction in fruit size was consistent with the observed decrease in fruit weight (Figure 5d; Figure S9d). Notably, sugar content remained relatively consistent across all genotypes, suggesting that the reductions in fruit weight and size in *slga3ox3*/*4* plants did not compromise fruit quality (Figure 5d; Figure S9d). Additionally, the fruit‐setting rate was reduced in both *slga3ox4* and *slga3ox3*/*4* mutants compared to triple‐determinate plants, further contributing to overall yield reduction (Figure 5d; Figure S9d).

**Figure 6:**
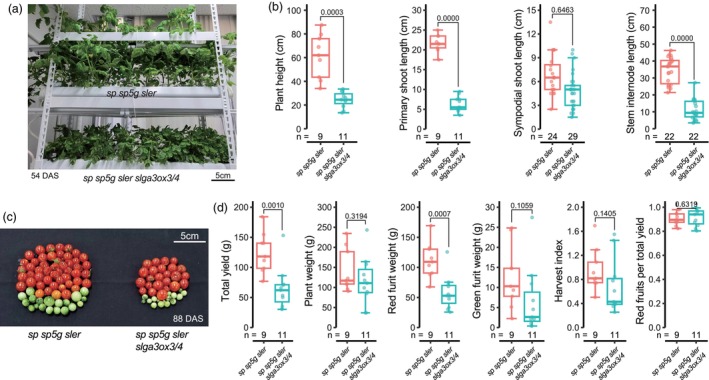
Plant architecture and yield performance of *slga3ox3/4* mutants in a vertical farming system. (a) The *sp sp5g sler* and *sp sp5g sler slga3ox3*/*4* cultivated in the vertical farm. (b) Plant height, primary shoot length, sympodial shoot length and stem internode length of *sp sp5g sler* and *sp sp5g sler slga3ox3/4*. Plant height, length between last inflorescence and 1st leaf; Primary shoots, between 1st inflorescence and 1st leaf of the primary shoot; Sympodial shoot, between 1st–2nd and 2nd–3rd inflorescences; Stem internode, internode between the 5th–6th and 6th–7th leaves; (c) Total fruits of *sp sp5g sler*, *sp sp5g sler slga3ox3*, *sp sp5g sler slga3ox4* and *sp sp5g sler slga3ox3/4*. (d) Total yield, plant weight, red fruit weight, green fruit weight, harvest index and red fruits per total yield of *sp sp5g sler* and *sp sp5g sler slga3ox3/4*. (b and d) Box plots, 25th–75th percentile; center line, median; whiskers, full data range. Exact sample sizes (*n*) for replicate types are indicated. Statistical analyses were performed using a two‐tailed, two‐sample *t*‐test. At least two experiments were repeated independently with similar results.

Despite the yield reduction, the compact growth of *slga3ox3/4* plants may offer advantages for vertical farming. In a controlled vertical farming system using nutrient film technique hydroponics, *slga3ox3/4* plants demonstrated a more optimal plant size than triple‐determinate plants, fitting well within the growing area (Figure 6a). In the *slga3ox3/4* mutant plants, the reduction in internode length led to a decrease in primary shoot length (Figure 6b). Although no significant differences were observed in sympodial shoot length, the reduction in primary shoot length significantly decreased overall plant height. This trend remained consistent when the plants were cultivated in greenhouse conditions. Both triple‐determinate and *slga3ox3*/*4* plants exhibited a yield decrease when cultivated in the vertical farm, as opposed to greenhouse conditions (Figure 6c,d). However, the yield reduction in *slga3ox3*/*4* plants was less pronounced compared to triple‐determinate plants. Specifically, triple‐determinate plants showed a reduction of 75% in yield when grown in the vertical farm, in contrast to greenhouse conditions, while *slga3ox3/4* mutants exhibited only a 37% decrease under the same conditions (Figures 5b and 6d). Notably, no significant variation was observed in either the harvest index or the proportion of red fruits to total yield (Figure 6d). These findings suggest that *slga3ox3/4* plants may be more suitable for high‐density planting configurations, facilitating improved cultivation efficiency.

## Discussion

In this study, we introduced three *slga3ox* mutations into *sp sp5g sler* triple‐determinate plants and evaluated the phenotypic differences of *slga3ox* single and double mutants. Given the unclear functional distinctions between *SlGA3ox* genes, our findings could illuminate their functional differences. The *Arabidopsis* orthologue of *SlER*, which is integral to the triple‐determinate tomato genotype, plays a key role in modulating GA signalling (Du *et al*., 2018). Our results showed that combining *slga3ox3* and *slga3ox4* mutations significantly enhances stem growth inhibition already present from *sler* mutations (Figures 2b,c and 3a,b; Figures S5–S7). However, the independent roles of *SlGA3ox3*, *SlGA3ox4* and *SlGA3ox5* in stem elongation could not be demonstrated in the absence of *sler*. Introducing other *slga3ox* mutations into *sp sp5g* double‐determinate plants will help pinpoint their individual effects and refine stem length customization. The expression of *SlGA3ox5* was minimal, and *slga3ox* single mutants showed no noticeable changes in shoot architecture (Figure 1d,e; Figure S3). However, introducing the *slga3ox5* mutation into the *slga3ox3*/*4* mutant background may produce an additive effect on shoot architecture due to the disruption of functionally redundant genes. Our findings indicate that *sp sp5g sler slga3ox3*/*4* mutants already possess a plant stature suitable for vertical farming applications. Therefore, rather than expanding gene editing to additional *SlGA3ox* members, it may be more beneficial to focus on strategies to alleviate the negative growth effects of *slga3ox3*/*4* mutants. Nevertheless, for future investigations into genetic redundancy within the SlGA3ox gene family, functional analysis could be further refined by generating and characterizing higher‐order mutants, such as *slga3ox3*/*4*/*5*.

We found that the gene‐edited tomato plants cultivated in cocopeat exhibited longer stem internode lengths compared to those grown in ebb and flow hydroponic systems, although the phenotypic trends among the various mutant lines remained consistent across both growth conditions (Figures 2 and 3; Figures S6 and S7). Notably, the *slga3ox3/4* double mutants exhibited more pronounced dwarfism than either the triple‐determinate, *slga3ox3* and *slga3ox4* plants, indicating the functional redundancy between *SlGA3ox3* and *SlGA3ox4* in regulating stem elongation. Physiological measurements demonstrated that *slga3ox3/4* plants showed lower photosynthetic efficiency (*F*_*v*_/*F*_*m*_) and stomatal conductance, coupled with higher leaf temperatures (Figure 4b; Figure S8c). The *F*_*v*_/*F*_*m*_, indicative of the maximum quantum efficiency of photosystem II, serves as a critical indicator of plant stress (Baker, 2008). The diminished stomatal conductance observed in *slga3ox3/4* plants correlates with elevated leaf temperatures, suggesting the additional roles of *SlGA3ox3* and *SlGA3ox4* beyond stem elongation. Taken together, *SlGA3ox3* and *SlGA3ox4* might be associated with plant adaptability to environmental stress.

An imbalance of plant hormones, including GA, is known to impact fruit development (Fenn and Giovannoni, 2021). In our study, both *slga3ox3* and *slga3ox4* single mutants exhibited a modest reduction in total yield per plant compared to triple‐determinate plants. This reduction was further amplified in the *slga3ox3*/*4* double mutants, primarily due to smaller fruit size, delayed ripening and lower fruit set (Figure 5b,d; Figure S9b,d). Additionally, the reduced yield in *slga3ox3*/*4* plants led to a slight decrease in the harvest index. Although overall plant size was reduced, the proportional decline in fruit yield resulted in either a marginally lower or similar harvest index (Figures 5b and 6d; Figure S9b). Given that a high harvest index is a crucial trait for vertical farming, enhancing the yield potential of *slga3ox3*/*4* plants will be essential to improve their suitability for vertical farming systems (Kwon, 2023; SharathKumar *et al*., 2020). To mitigate these negative phenotypic outcomes and customize the desired traits, *cis*‐regulatory regions of these genes can be edited using CRISPR technology (Zhou *et al*., 2023). Precise editing of coding and non‐coding genomic regions offers a strategy to fine‐tune protein function and gene expression (Glaus *et al*., 2025; Lanctot *et al*., 2025; Lou *et al*., 2025). Additionally, integrating gene mutations that influence fruit size or enhance ripening might compensate for these drawbacks (Gapper *et al*., 2013). It will be necessary to understand the sink‐source relationship and nutrient distribution, as the *slga3ox3/4* double mutants show a drastic reduction in shoot biomass, which may lead to a decrease in total photosynthesis, limiting the ability to increase the productivity of individual plants (Lemoine *et al*., 2013).

Further studies could benefit from targeting additional GA biosynthetic enzymes to develop new plant varieties better suited for vertical farming environments. Specifically, integrating mutations in *SlGA3ox* and *SlGA20ox* genes, key players of bioactive GA synthesis, may offer new avenues to modify plant size in tomato plants (Plackett *et al*., 2012). Investigating various *SlGA3ox* and *SlGA20ox* genes could reveal distinct functional differences that could be exploited to fine‐tune plant architecture effectively (Plackett *et al*., 2012; Wang *et al*., 2017). For instance, the disruption of the *SlGA3ox5*, noted for its minimal expression, did not induce noticeable phenotypic changes (Figures 1d,e and 2e,f; Figure S3). However, its combination with other *SlGA3ox* and *SlGA20ox* genes could lead to significant modifications in agronomic traits. Advanced genetic editing techniques could also be applied to both the coding and non‐coding regions of these enzymes to adjust the synthesis of bioactive GA, providing precise control over critical growth parameters such as stem length and overall plant stature (Chen *et al*., 2023). This strategy has the potential to revolutionize the cultivation of various crops, including fruits, medicinal plants, specialty high‐value crops, cosmetic plants, and even small trees, significantly impacting the development of controlled environment agriculture (Benke and Tomkins, 2017; O'Sullivan *et al*., 2019).

Given the reduced yield observed in *ga3ox3*/*4* double mutants, it is essential to investigate approaches that can enhance overall productivity. Alongside the genetic enhancements previously discussed, the productivity challenges of *slga3ox3/4* double mutants can also be addressed by refining cultivation practices (SharathKumar *et al*., 2020). An effective approach includes the application of exogenous GA sprays after flowering, aimed at boosting fruit yield (Bünger‐Kibler and Bangerth, 1982). While the yield per individual plant of *slga3ox3/4* plants is lower, the overall yield per unit area can be enhanced through high‐density planting, leveraging the vertical farming setup (Kwon, 2023; Kwon *et al*., 2020). This speculation is supported by the observation that individual slga3ox3/4 plants exhibited improved yield potential under vertical farming conditions compared to cocopeat‐based cultivation (Figures 5 and 6; Figure S9). Given the perpetual cycle of planting and harvesting inherent to vertical farming, it is imperative to maintain optimal productivity per unit area consistently throughout the year (Kwon, 2023; van Delden *et al*., 2021). It is critical to optimize plant size and architecture to facilitate effective cultivation and harvesting in the distinct layers of a vertical farm, especially if the individual plant productivity is hindered by poor fertility. In addition to crop improvement, tailor‐made cultivation strategies for newly bred plants are crucial to maximal production in vertical farm systems. This requires precise calculations on the optimal number of plants per area, the ideal planting density and the number of layers in the growth space to ensure maximum productivity per unit area.

Consequently, this comprehensive approach ensures a systematic examination and confirmation of the stability of gene‐edited crops across various growing environments. This establishes a strong foundation for their potential use in agriculture, particularly in constrained spaces such as vertical farms. This thorough validation process not only reinforces the reliability of our genetic modifications but also opens pathways for optimizing crop production in diverse agricultural environments.

## Materials and methods

### Plant materials and growth conditions

The tomato cultivar ‘Sweet100’ seeds used in this study were sourced from our own stocks. Seeds were sown directly into rockwool and grown in a greenhouse at Kyung Hee University in Yongin, Republic of Korea, following established methods (Lim *et al*., 2024). The nutrient solution had an average pH of 5.8 and was replenished every two days. Transplantation onto cocopeat or ebb and flow beds occurred between 28 and 40 days post‐sowing. In the ebb and flow system, various hydroponic fertilizers were used to ensure the plants received sufficient nutrients (Lim *et al*., 2024). Plants grown in cocopeat were watered via drip irrigation and received regular fertilizer applications. Electrical conductivity (EC) was gradually adjusted from 1.0 to 2.2 dS/m. Throughout the study, tomato plants were cultivated in the greenhouse under long‐day conditions, supplemented with artificial light from high‐pressure sodium bulbs (Lim *et al*., 2024). Detailed information about the cultivation environment can be found in Table S1 of the manuscript. Plants showing signs of disease or damage were excluded from the data analysis.

### Gene editing and plant transformation

CRISPR‐Cas9 targeted mutagenesis and plant transformation techniques were utilized for editing the tomato genome, following established procedures (Brooks *et al*., 2014; Kwon *et al*., 2020). In brief, guide RNAs (gRNAs) were designed using CRISPRdirect software (https://crispr.dbcls.jp/), and binary vectors were constructed via Golden Gate cloning, following described protocols (Naito *et al*., 2015; Werner *et al*., 2012). *SlGA3ox3* and *SlGA3ox4* were simultaneously targeted, with three sgRNAs designed for each gene, resulting in a total of six sgRNAs. Additionally, three sgRNAs were selected for the gene editing of *SlGA3ox5*. Genomic DNA was isolated from at least three independent leaf tissues per T_0_ plant to confirm transgene insertions and CRISPR‐induced mutations, as previously outlined (Kwon *et al*., 2022). The *SlGA3ox3*, *SlGA3ox4* and *SlGA3ox5* genes were targeted by the system, with transgenic T_0_ plants subsequently transferred to soil for genotyping validation of CRISPR‐induced mutations using PCR and Sanger sequencing, following established methodologies. Detailed primer sequences and guide RNA information are provided in Table S4.

### Plant phenotyping and imaging

Phenotypic characterization was conducted with plants from which the inserted vector had been removed through generational advancement from T_0_ plants. In detail, transgene‐free plants harbouring homozygous mutations were identified and selected from the T_1_ and F_1_ generations. Phenotypic analyses were performed using plants from the T_2_ and F_2_ generations and their offspring. Plants were sprayed with 400 mg/L kanamycin and genotyped by PCR to confirm the absence of the Cas9 coding gene sequences. All phenotypic quantification procedures in this study were conducted according to previously described methods (Kwon *et al*., 2022). In summary, phenotypic characterization involved non‐transgenic homozygous plants obtained through backcrossing or self‐pollination. To verify the absence of transgenes and CRISPR‐edited DNA sequences, all tomato mutant plants were treated with kanamycin and genotyped using specific primers (Table S4). Pedicels, peduncles and inflorescence internodes were manually measured when at least half of the flowers had opened in the inflorescences. Shoot lengths and heights were evaluated using standard 50‐cm and 3‐m rulers. Data on the number of flowers, inflorescences and leaves per sympodial shoot were collected from plants and inflorescences with the corresponding stage of development. For the analysis of the number of leaves per sympodial shoot, leaf numbers on the main shoot were counted before the initiation of the next inflorescence, as described previously. The exact numbers of individuals (*n*) are presented in all figures, and the exact sample numbers for quantification are indicated in both figures and Tables S5–S7, S10, S11, and S14.

### Yield trials under greenhouse conditions

Tomato yield trials were conducted as described previously with some modifications (Lim *et al*., 2024). The trials for *sp sp5g sler*, *sp sp5g sler slga3ox3*, *sp sp5g sler slga3ox4* and *sp sp5g sler slga3ox3/4* were carried out twice on greenhouse‐grown plants. All axillary shoots, except for the first one, were removed. Yield evaluation was based on the fruits produced from the main shoot and the basal axillary shoot. Four cocopeat blocks were used, with ten plants planted per block (providing a cultivation area of 200 cm^2^ per plant). Breaker fruits were classified as red fruits. The total fruit yield was calculated by summing the green and red fruits from each plant. Harvest indices were calculated by dividing the total fruit weight by the plant biomass. Fruit mass was quantified using a digital scale (HANSUNG). Size and mass measurements of mature red fruits were performed using an electronic digital calliper (Mitutoyo) and a digital scale (HANSUNG), respectively. Sugar content in fruit juice was assessed by measuring the Brix value (percentage) using a digital Brix refractometer (ATAGO). The fruit‐setting rate was calculated as the ratio of the total number of red fruits per plant to the total number of flowers per plant. The number of red fruits was estimated by dividing the total red fruit weight by the average weight of individual red fruits. To determine the total flower count, the average number of flowers from three representative inflorescences was multiplied by the total number of inflorescences on both the main shoot and basal axillary shoots. The exact numbers of individuals (*n*) are presented in all figures, and the exact sample numbers for quantification are indicated in both figures and Tables S9, S13 and S14.

### Growth conditions of hydroponic vertical farm

To grow *sp sp5g sler* and *sp sp5g sler slga3ox3/4* in a hydroponic vertical farm, seeds were sown in rockwool in flats with 40‐cell plastic inserts. After sowing, tomato plants were cultivated in the greenhouse at Kyung Hee University under long‐day conditions (16 h of light, 26–28 °C/8 h of darkness, 18–20 °C; 40–60% relative humidity), supplemented with artificial light from high‐pressure sodium bulbs. Seedlings were transplanted onto the hydroponic vertical farm indoors at Kyung Hee University between 30 and 35 days after sowing. In the vertical farm, each layer measured 25 cm in height, 90 cm in width and 30 cm in length, with 11 plants per layer. Automated irrigation systems, such as the nutrient film technique, were operated with fertilizer on a 15‐min on/165‐min off cycle. EC gradually increased from 0.7 to 1.3 dS/m. Plants in the columns were grown with artificial light from a red/blue LED (450 μmol/m^2^/s) and the long‐day conditions (16 h of light, 24–26 °C/8 h of darkness, 20–22 °C; 40–60% relative humidity).

### Measurement of photosynthetic pigments

Stomatal conductance to water vapour (*g*_sw_), Chlorophyll a fluorescence‐derived *F*_*v*_ */F*_*m*_ (optimal quantum yield efficiency of photosystem II) and leaf temperature (*T*_leaf_) were measured using a LI‐600 (LI‐COR). Leaf chlorophyll index was measured using both non‐destructive and destructive assays. The non‐destructive leaf chlorophyll index assay was measured using a SPAD meter (Konica Minolta). Photosynthetic pigments were measured in terminal leaflets, and tissues from the 7th and 9th leaves were collected. To assess total chlorophyll and carotenoid levels, leaves were extracted using an 80% acetone solution. Pigment concentrations were quantified using a UV/VIS spectrophotometer (Agilent) and calculated as previously described (Lichtenthaler and Buschmann, 2001). The exact numbers of individuals (*n*) are presented in all figures, and the exact sample numbers for quantification are indicated in both figures and Tables S8 and S12.

### Germination rate test

Germination was evaluated by placing seeds on moistened filter papers in petri dishes, which were then incubated at 28 °C in complete darkness. Each treatment consisted of three biological replicates, with 20 seeds per replicate.

### Gene annotation and accession numbers

Sequence data for tomato is derived from the Sol Genomics Network (https://solgenomics.net; SL4.0). *SlGA3ox1*, *Solyc06g066820. SlGA3ox2*, *Solyc03g119910. SlGA3ox3*, *Solyc01g058250. SlGA3ox4*, *Solyc05g052740. SlGA3ox5*, *Solyc02g038808*. Accession numbers from other species are provided in Table S2.

### Phylogenetic and gene expression analyses

The amino acid sequences for tomato, potato, eggplant, and pepper were extracted from the Sol Genomics Network (https://solgenomics.net). The peptide sequence data for groundcherry are derived from the groundcherry genome assembly database (https://github.com/pan‐sol/pan‐sol‐data/tree/main/Physalis) (He *et al*., 2023). Coding sequence data for *Arabidopsis* and rice were obtained from TAIR (https://www.arabidopsis.org/) and Phytozome (https://phytozome‐next.jgi.doe.gov/), respectively. We use MEGA‐X software (https://www.megasoftware.net/) to construct a comparative phylogenetic tree by the Maximum Likelihood Estimation (MLE) method. Bootstrap values from 1000 replicates are presented on each node. We performed motif analysis of protein sequences using MEME (https://meme‐suite.org/meme/index.html). We conducted multiple alignments using Clustal Omega (https://www.ebi.ac.uk/Tools/msa/‐clustalo/). To investigate gene expression patterns, we utilized CAFRI‐Tomato (https://cafri.khu.ac.kr/tomato/) and the Rose Lab Atlas in the Tomato eFP Browser (https://bar.utoronto.ca/efp_tomato/cgi‐bin/efpWeb.cgi) (Hong *et al*., 2020). We utilized TBtools to depict gene structure in our study (Chen *et al*., 2020). The information regarding the normalized counts of RNA‐seq used in this study can be found in Table S3.

### RNA extraction, complementary DNA synthesis and quantitative real‐time PCR

Total RNA was extracted from triple‐determinate plants using established protocols with minor modifications, and gene expression was subsequently quantified by quantitative real‐time PCR (qPCR) (Kwon *et al*., 2022). Briefly, total RNA was isolated from tissue samples using both the RNeasy Plant Mini Kit (Qiagen) and the PURE™ Plant RNA Extraction Kit (Infusion Tech), following the manufacturers' protocols. For cDNA synthesis, 1 μg of total RNA was reverse‐transcribed with the iScript cDNA Synthesis Kit (Bio‐Rad). Quantitative real‐time PCR (qPCR) was then performed using gene‐specific primers (Table S4) in conjunction with the iQ SYBR Green Supermix (Bio‐Rad) on a CFX96 Real‐Time PCR Detection System (Bio‐Rad). For cotyledon, meristem, stem, leaf, flower and green fruit tissues, five biological replicates were used, whereas seven biological replicates were employed for roots. Each biological replicate was analysed in four technical replicates.

### Statistical analyses

Statistical calculations were conducted using R (RStudio (v.2022.12.0 + 353)), Microsoft Excel and ANOVA Calculator (https://www.statskingdom.com/180Anova1way.html#R). Statistical analyses were performed using a one‐way analysis of variance (ANOVA) with a Tukey test and a two‐tailed, two‐sample *t*‐test.

## Author contributions

Y.L. performed the experiments, prepared the figures and wrote the manuscript. M.‐G.S., J.L., S.H., J.‐T.A. and H.‐Y.J. performed the tomato experiments and phenotypic characterization. W.‐J.H. performed the phylogenetic and transcriptome analyses. H.‐I.C. and C.L. helped perform the experiments. S.J.P. generated transgenic tomato plants and performed the tomato experiments. C.‐T.K. conceived the research, supervised and performed the experiments, prepared the figures and wrote the manuscript. All authors read and approved the manuscript.

## Conflict of interest

The authors declare that they have no conflicts of interest.

## Supporting information


**Figure S1** The process of gene editing to optimize tomato size and architecture for vertical farming.
**Figure S2** Gene and protein sequence analyses of *SlGA3ox*.
**Figure S3** Expression of *SlGA3ox* genes across multiple tomato tissues.
**Figure S4** Structure and protein sequence of mutant forms of SlGA3ox proteins.
**Figure S5** Shoot and inflorescence architecture of *slga3ox3*, *slga3ox4* and *slga3ox3/4* mutants in the ebb and flow beds.
**Figure S6** CRISPR targeted mutagenesis of *SlGA3ox3* and *SlGA3ox4* genes.
**Figure S7** Shoot and inflorescence architecture of *slga3ox3*, *slga3ox4* and *slga3ox3/4* mutants.
**Figure S8** Physiological analysis of *slga3ox3*, *slga3ox4* and *slga3ox3/4* mutants.
**Figure S9** Yield potential analysis of *slga3ox3*, *slga3ox4* and *slga3ox3/4* mutants.

**Table S1** Detailed environmental conditions in the greenhouse.
**Table S2** Protein sequences of GA3ox homologues for phylogenetic analysis.
**Table S3** Normalized counts from RNA‐seq and qPCR analysis in this study.
**Table S4** Primer and guide RNA sequences for this study.
**Table S5** Length quantification data of shoots and inflorescences in this study (Related to Figure 2d and Figure S6b,c).
**Table S6** Length quantification data of shoots and inflorescences in this study (Related to Figure 2f).
**Table S7** Length quantification data of shoots and inflorescences in this study (Related to Figure 3b,c,e).
**Table S8** Physiological analysis data in this study (Related to Figure 4b,c).
**Table S9** Yield potential analysis data in this study (Related to Figure 5b,d).
**Table S10** Length quantification data of shoots and inflorescences in this study (Related to Figure S5b and c).
**Table S11** Length quantification data of shoots and inflorescences in this study (Related to Figure S7c,d,f).
**Table S12** Physiological analysis data in this study (Related to Figure S8b–f).
**Table S13** Yield potential analysis data in this study (Related to Figure S9b,d).
**Table S14** Length quantification data of shoots in this study (Related to Figure 6b,d).

## Data Availability

All datasets generated in this study are included in the article and Supporting Information. The Sanger sequencing data for CRISPR‐generated sequences are in Supporting Information.
