# Supplementary material for: Optimizing plant size for vertical farming by editing stem length regulators
Source: Plant Biotechnol J. 2025 May 9;23(8):3041–53. doi: 10.1111/pbi.70129 (PMC12310881; doi:10.1111/pbi.70129)
Supplement: Supplementary file 1 — Figure S1 The process of gene editing to optimize tomato size and architecture for vertical farming. Figure S2 Gene and protein sequence analyses of SlGA3ox. Figure S3 Expression of SlGA3ox genes across multiple tomato tissues. Figure S4 Structure and protein sequence of mutant forms of SlGA3ox proteins. Figure S5 Shoot and inflorescence architecture of slga3ox3, slga3ox4 and slga3ox3/4 mutants in the ebb and flow beds. Figure S6 CRISPR targeted mutagenesis of SlGA3ox3 and SlGA3ox4 genes. Figure S7 Shoot and inflorescence architecture of slga3ox3, slga3ox4 and slga3ox3/4 mutants. Figure S8 Physiological analysis of slga3ox3, slga3ox4 and slga3ox3/4 mutants. Figure S9 Yield potential analysis of slga3ox3, slga3ox4 and slga3ox3/4 mutants. [file PBI-23-3041-s001.pdf]

Supplementary Figures

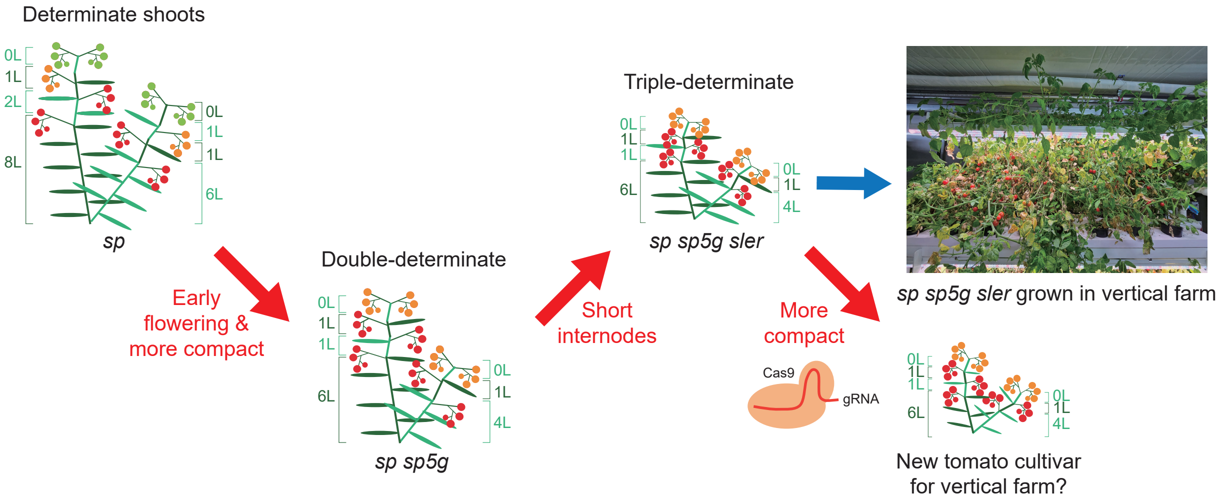

Figure S1. The process of gene editing to optimize tomato size and architecture for vertical farming. The tomato diagrams were adapted and modified from previous study (Kwon et al., 2020).

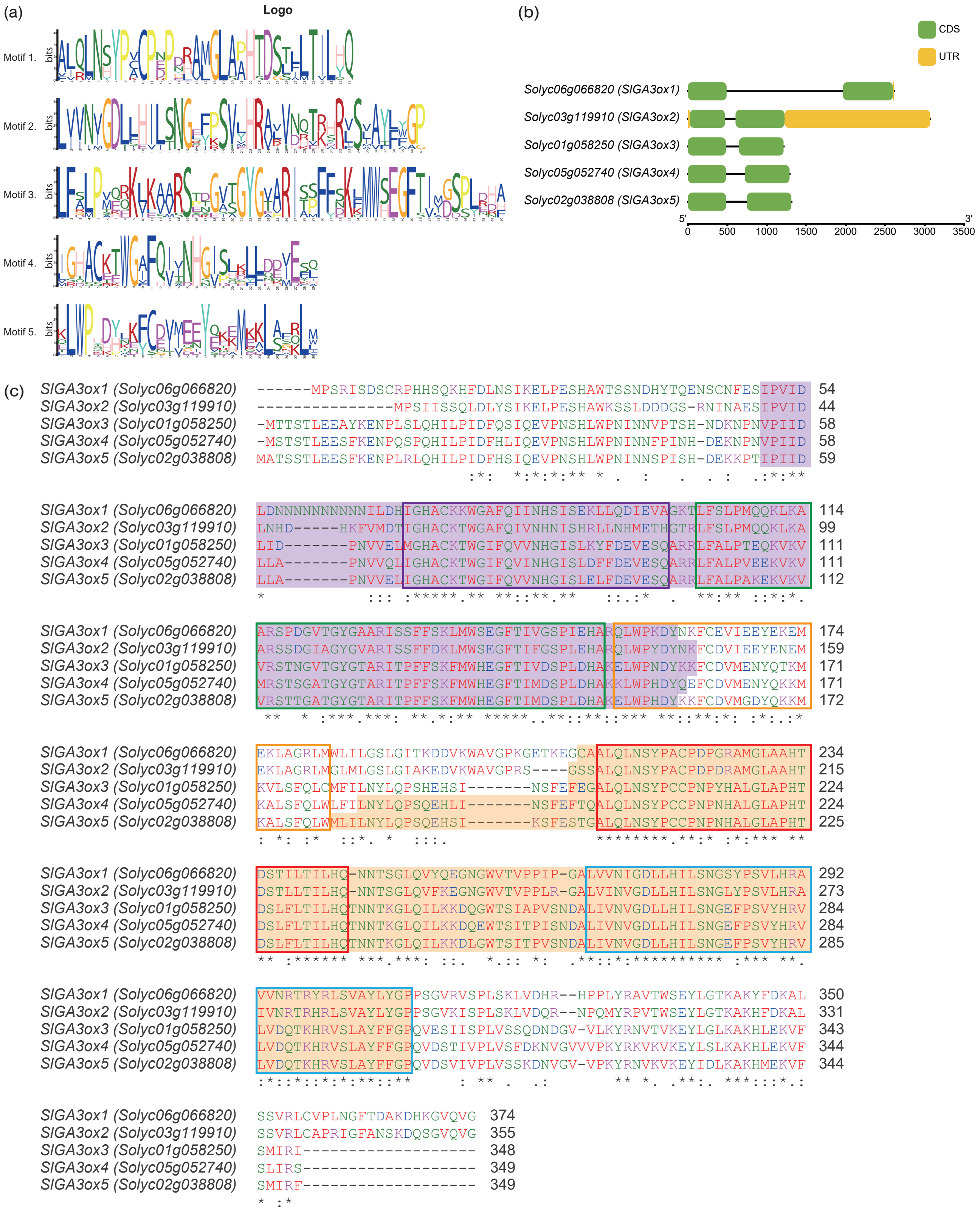

Figure S2. Gene and protein sequence analyses of *SlGA3ox*. (a) Motif analysis of SlGA3ox protein sequences using MEME. (b) Gene structure of *SlGA3ox* genes using TBtools. (c) Multiple alignments of SlGA3ox proteins using Clustal Omega. Purple boxes indicate the DIOX (non-haem dioxygenase in morphine synthesis N-terminal) domain region. Orange boxes indicate the 2OG oxygenase (2OG-Fe (II) oxygenase superfamily) domain region.

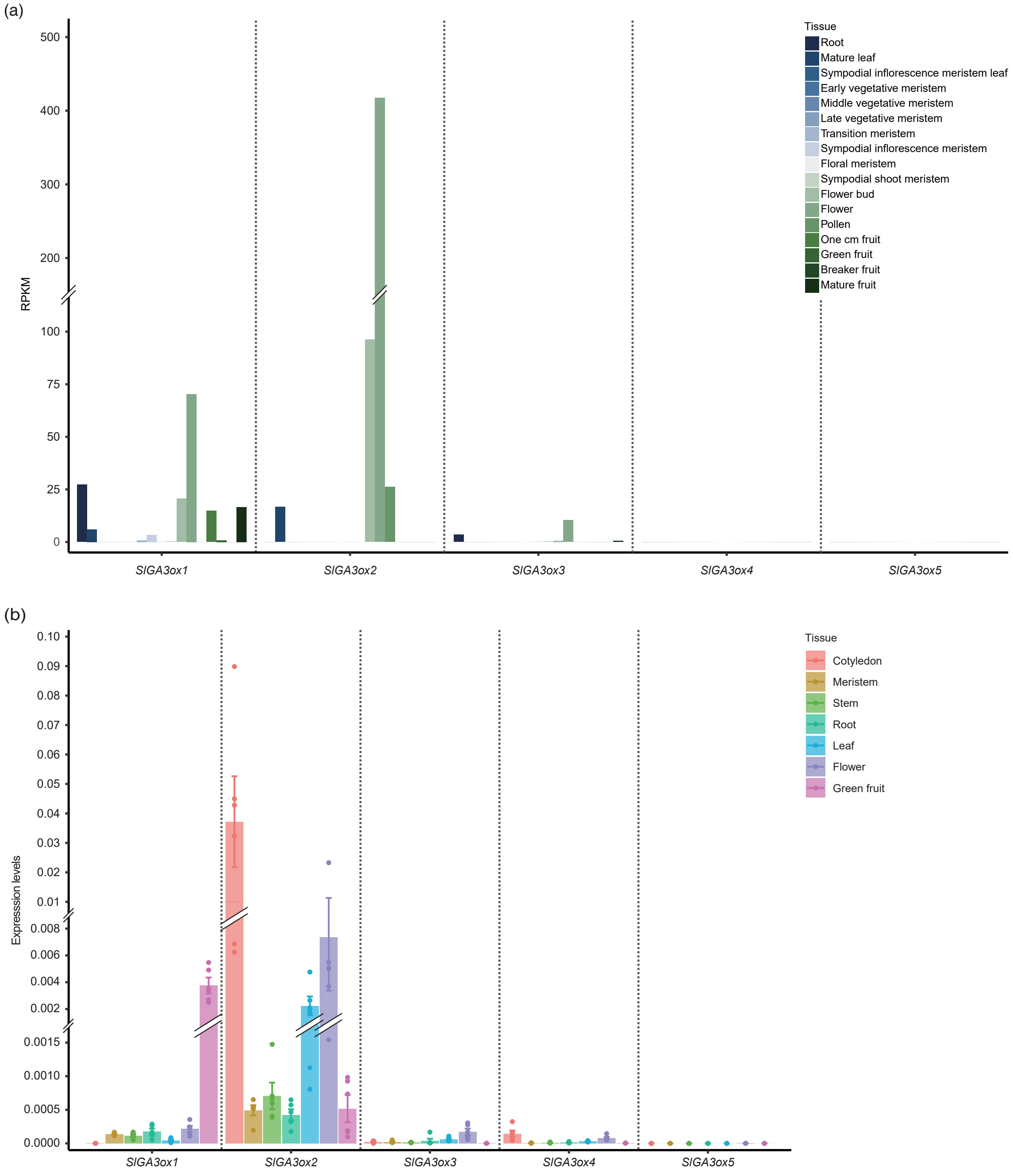

Figure S3. Expression of *SlGA3ox* genes across multiple tomato tissues. (a) Normalized expression levels of five *SlGA3ox* genes based on RNA-seq data. RPKM, reads per kilobase of transcript per million mapped reads. (b) qRT-PCR analysis of *SlGA3ox* gene expression in cotyledon, meristem, stem, root, leaf, flower, and green fruit tissues of triple-determinate plants. Expression levels were normalized to tomato *Ubiquitin3* (*SlUBQ3*).

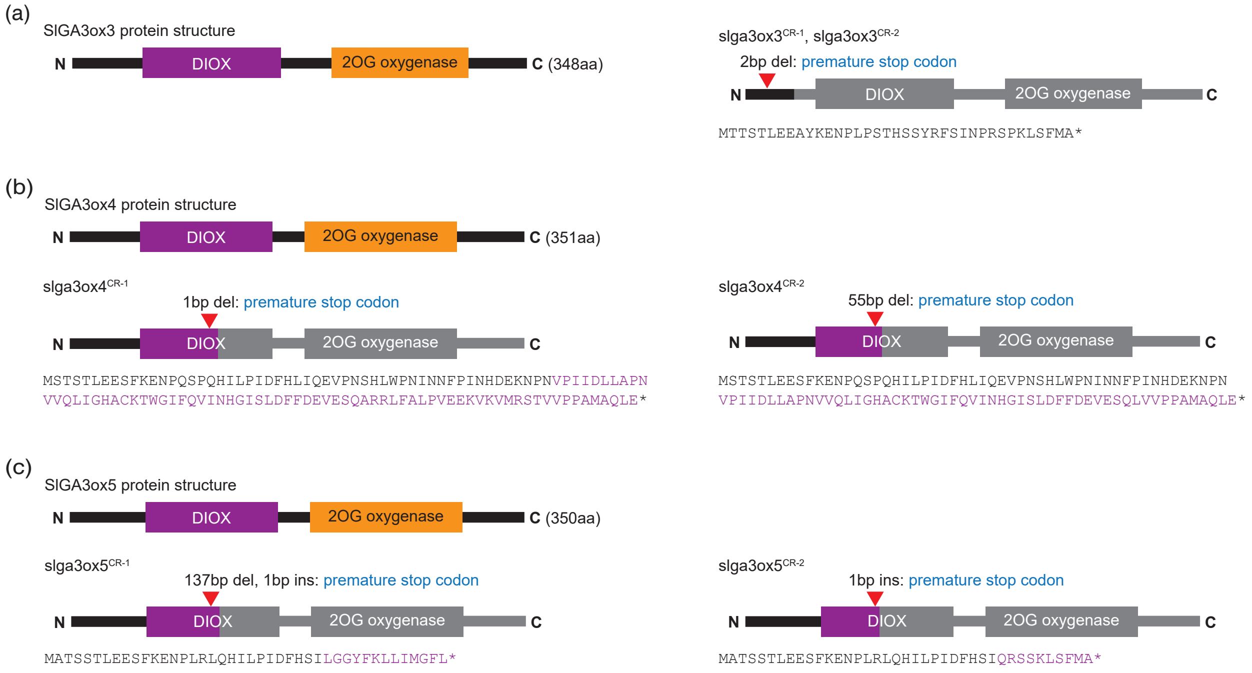

Figure S4. Structure and protein sequence of mutant forms of SlGA3ox proteins. (a) SlGA3ox3 protein models of wild-type, *slga3ox3*$^{CR\text{-}1}$, and *slga3ox3*$^{CR\text{-}2}$. (b) SlGA3ox4 protein models of wild-type, *slga3ox4*$^{CR\text{-}1}$, and *slga3ox4*$^{CR\text{-}2}$. (c) SlGA3ox5 protein models of wild-type, *slga3ox5*$^{CR\text{-}1}$, and *slga3ox5*$^{CR\text{-}2}$. (a–c) Protein domain sites are highlighted in purple and orange. Gray lines and rectangles represent untranslational protein sequence by premature stop codons.

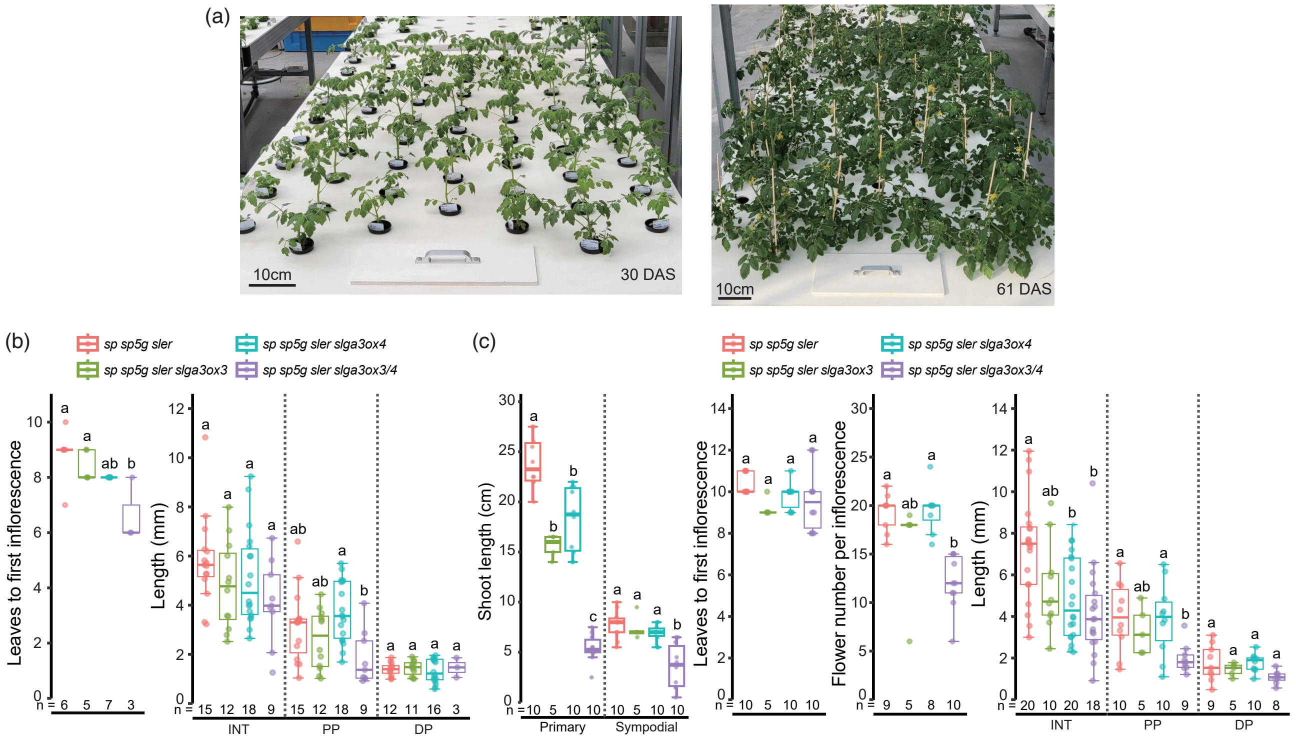

Figure S5. Shoot and inflorescence architecture of *slga3ox3*, *slga3ox4*, and *slga3ox3/4* mutants in the ebb and flow beds. (a) Tomato plants grown on the ebb and flow beds. (b) Leaves to first inflorescence, INT, PP, and DP length of *sp sp5g sler, sp sp5g sler slga3ox3, sp sp5g sler slga3ox4,* and *sp sp5g sler slga3ox3/4*. The plants grown from May 09, 2023 to July 01, 2023. INT, internode between 1st–2nd, 2nd–3rd, and 3rd–4th flowers of 2nd inflorescence; PP, proximal section of 1st–3rd pedicels; DP, distal section of 1st–3rd pedicels; (c) Shoot length of main shoots, leaves to first inflorescence, flower number per inflorescence, INT, PP, and DP length of *sp sp5g sler, sp sp5g sler slga3ox3, sp sp5g sler slga3ox4,* and *sp sp5g sler slga3ox3/4*. The plants grown from January 06, 2023 to March 29, 2023. Primary shoots, between 1st inflorescence and 1st leaf of the primary shoot; Sympodial shoot, between 1st and 2nd inflorescences; INT, internode between 1st–2nd and 2nd–3rd flowers; PP, proximal section of 1st pedicels; DP, distal section of 1st pedicels; (b and c) Box plots, 25th–75th percentile; center line, median; whiskers, full data range. Exact sample sizes (n) for replicate types are indicated. Letters indicate significance groups at $P < 0.05$ (one-way ANOVA and Tukey HSD). Different letters between genotypes indicate significance. At least twice experiments were repeated independently with similar results.

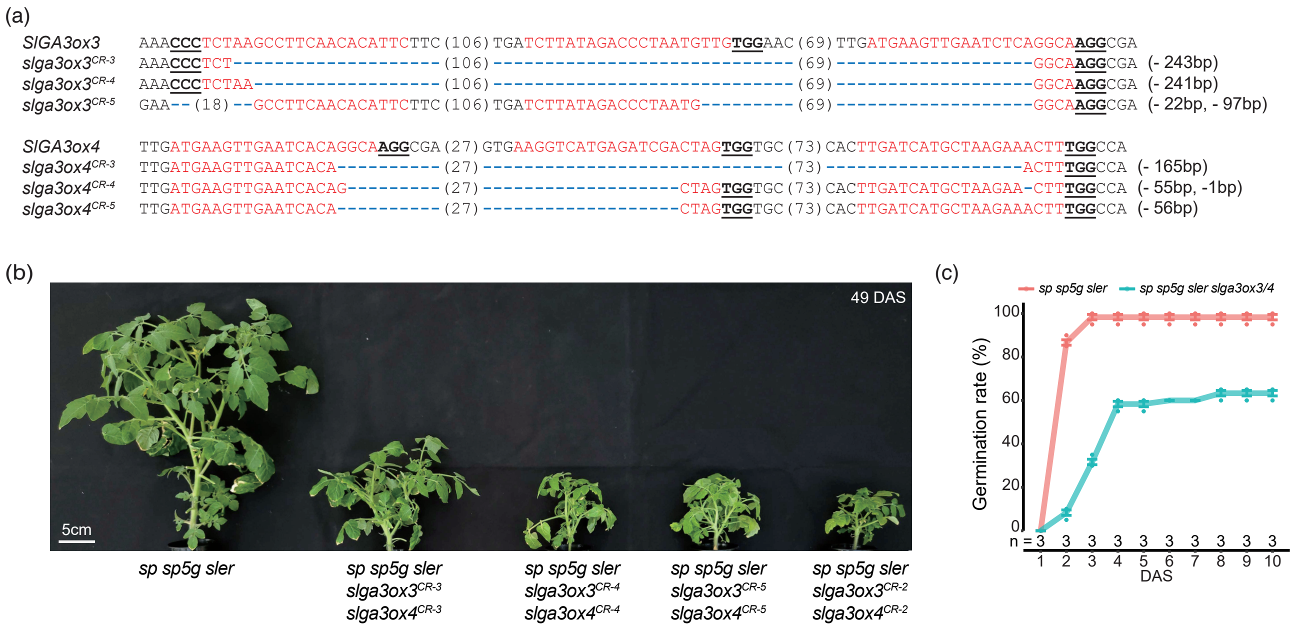

Figure S6. CRISPR targeted mutagenesis of *SlGA3ox3* and *SlGA3ox4* genes. (a) Sequences of *SlGA3ox3* and *SlGA3ox4* mutant alleles generated by CRISPR-Cas9. Guide RNA and protospacer-adjacent motif (PAM) sequences are highlighted in red and bold underlined, respectively. Blue dash and letter indicate deletion and insertion. Numbers in parentheses indicate gap lengths. (b) Shoots of *sp sp5g sler, sp sp5g sler slga3ox3*$^{CR\text{-}3}$ *slga3ox4*$^{CR\text{-}3}$, *sp sp5g sler slga3ox3*$^{CR\text{-}4}$ *slga3ox4*$^{CR\text{-}4}$, *sp sp5g sler slga3ox3*$^{CR\text{-}5}$ *slga3ox4*$^{CR\text{-}5}$, and *sp sp5g sler slga3ox3*$^{CR\text{-}2}$ *slga3ox4*$^{CR\text{-}1}$. DAS, days after sowing. (c) Germination analysis of *sp sp5g sler* and *sp sp5g sler ga3ox3/4*.

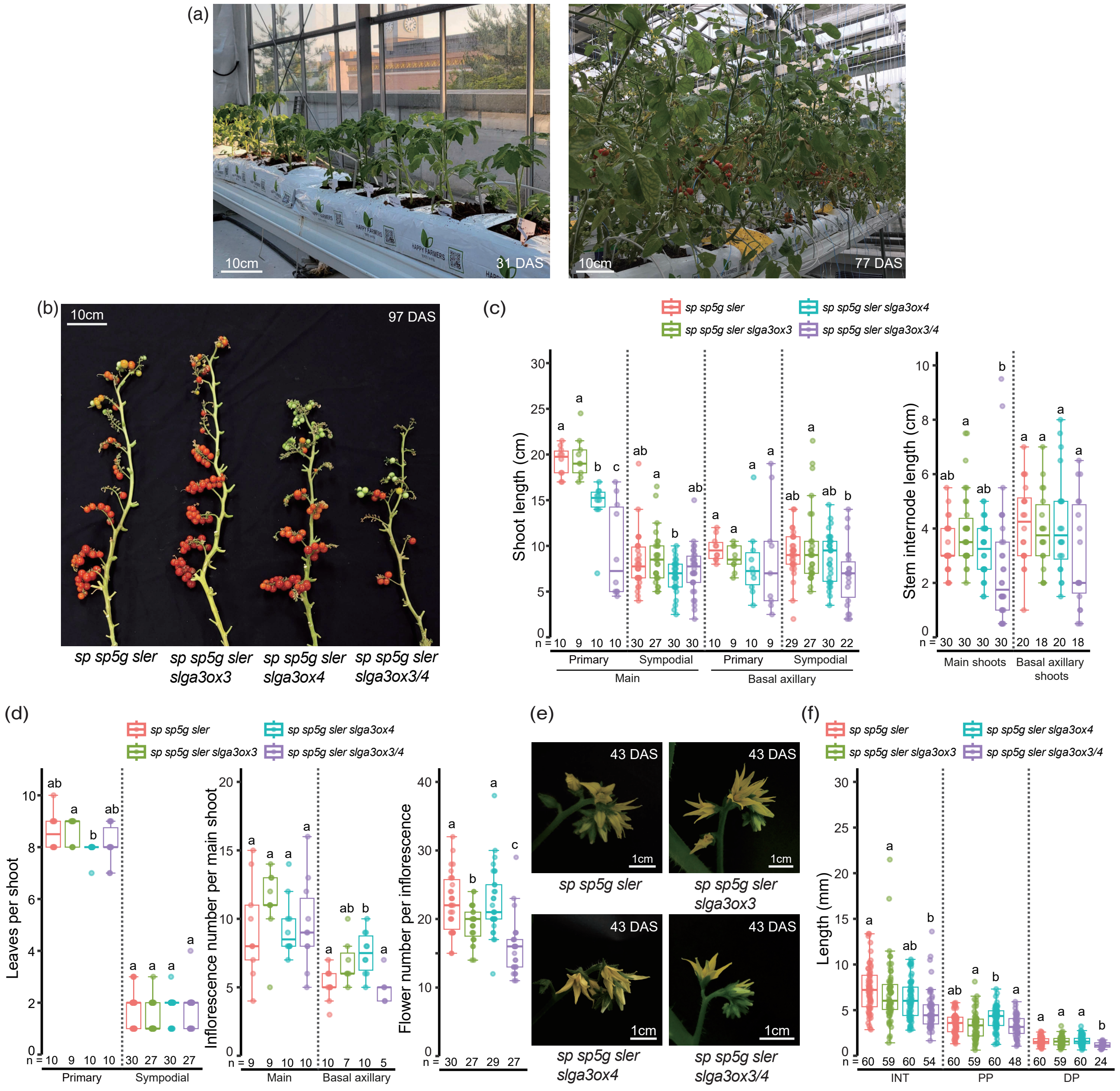

Figure S7. Shoot and inflorescence architecture of *slga3ox3, slga3ox4,* and *slga3ox3/4* mutants. (a) Tomato plants grown on cocopeat slabs. (b) Shoots of *sp sp5g sler, sp sp5g sler slga3ox3, sp sp5g sler slga3ox4*, and *sp sp5g sler slga3ox3/4*. (c) Shoot length and stem internode length of *sp sp5g sler, sp sp5g sler slga3ox3, sp sp5g sler slga3ox4,* and *sp sp5g sler slga3ox3/4*. Primary shoots, between 1st inflorescence and 1st leaf of the primary shoot; Sympodial shoot, between 1st–2nd, 2nd–3rd, and 3rd–4th inflorescences; Stem internode, internode between the 5th–6th, 6th–7th, and 7th–8th leaves of primary shoot; Stem internode of basal axillary shoot, internode between the 4th–5th and 5th–6th leaves of primary shoot; (d) Leaves per shoot, inflorescence number per main shoot, number of flowers per inflorescence of *sp sp5g sler, sp sp5g sler slga3ox3, sp sp5g sler slga3ox4,* and *sp sp5g sler slga3ox3/4*. (e) 2nd inflorescence of *sp sp5g sler, sp sp5g sler slga3ox3, sp sp5g sler slga3ox4,* and *sp sp5g sler slga3ox3/4*. (f) INT, PP, and DP length of *sp sp5g sler, sp sp5g sler slga3ox3, sp sp5g sler slga3ox4,* and *sp sp5g sler slga3ox3/4*. INT, inflorescence internode between 1st–2nd, 2nd–3rd, and 3rd–4th flowers of 2nd inflorescence; PP, proximal section of 1st–3rd pedicels; DP, distal section of 1st–3rd pedicels. (b–f) The plants grown from May 09, 2023 to August 14, 2023. (c, d and f) Box plots, 25th–75th percentile; center line, median; whiskers, full data range. Exact sample sizes (n) for replicate types are indicated. Letters indicate significance groups at $P < 0.05$ (one-way ANOVA and Tukey HSD). Different letters between genotypes indicate significance. At least twice experiments were repeated independently with similar results.

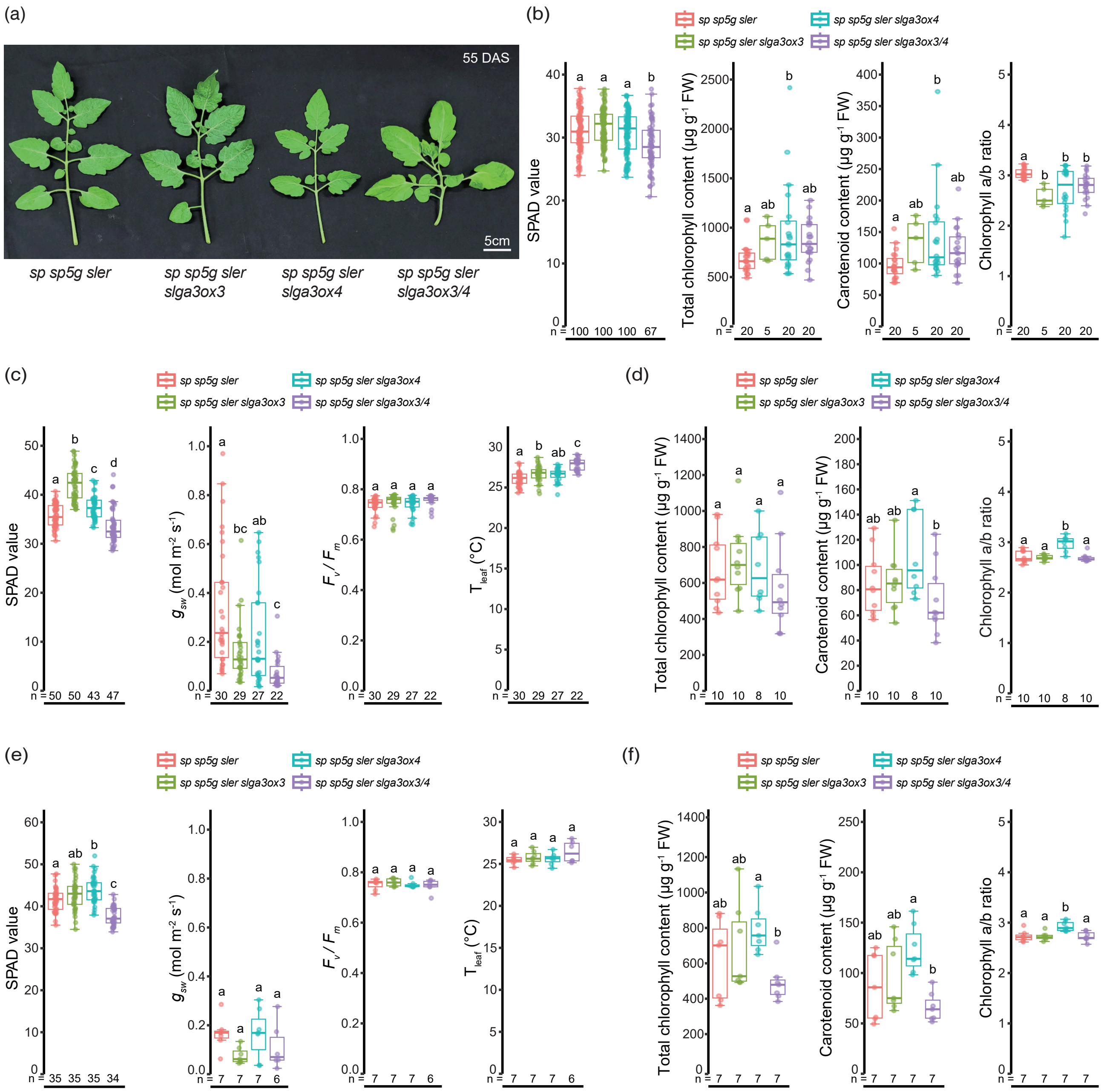

Figure S8. Physiological analysis of *slga3ox3, slga3ox4*, and *slga3ox3/4* mutants. (a) 7th leaf of *sp sp5g sler, sp sp5g sler slga3ox3, sp sp5g sler slga3ox4,* and *sp sp5g sler slga3ox3/4*. (b) SPAD value, total chlorophyll content, carotenoid content, and chlorophyll a/b ratio in *sp sp5g sler, sp sp5g sler slga3ox3, sp sp5g sler slga3ox4,* and *sp sp5g sler slga3ox3/4*. (c) SPAD value, g_{sw}, F_v/F_m, and T_{leaf} of *sp sp5g sler, sp sp5g sler slga3ox3, sp sp5g sler slga3ox4,* and *sp sp5g sler slga3ox3/4*. (d) Total chlorophyll content, carotenoid content, and chlorophyll a/b ratio in *sp sp5g sler, sp sp5g sler slga3ox3, sp sp5g sler slga3ox4,* and *sp sp5g sler slga3ox3/4*. (e) SPAD value, g_{sw}, F_v/F_m, and T_{leaf} of *sp sp5g sler, sp sp5g sler slga3ox3, sp sp5g sler slga3ox4,* and *sp sp5g sler slga3ox3/4*. (f) Total chlorophyll content, carotenoid content, and chlorophyll a/b ratio in *sp sp5g sler, sp sp5g sler slga3ox3, sp sp5g sler slga3ox4,* and *sp sp5g sler slga3ox3/4*. (a and b) The plants grown on cocopeat from May 09, 2023 to August 14, 2023. (c and d) The plants grown on the ebb and flow beds from May 09, 2023 to July 01, 2023. (e and f) The plants grown on the ebb and flow beds from January 06, 2023 to March 29, 2023. (b–f) Box plots, 25th–75th percentile; center line, median; whiskers, full data range. Exact sample sizes (n) for replicate types are indicated. Letters indicate significance groups at $P < 0.05$ (one-way ANOVA and Tukey HSD). Different letters between genotypes indicate significance. At least twice experiments were repeated independently with similar results.

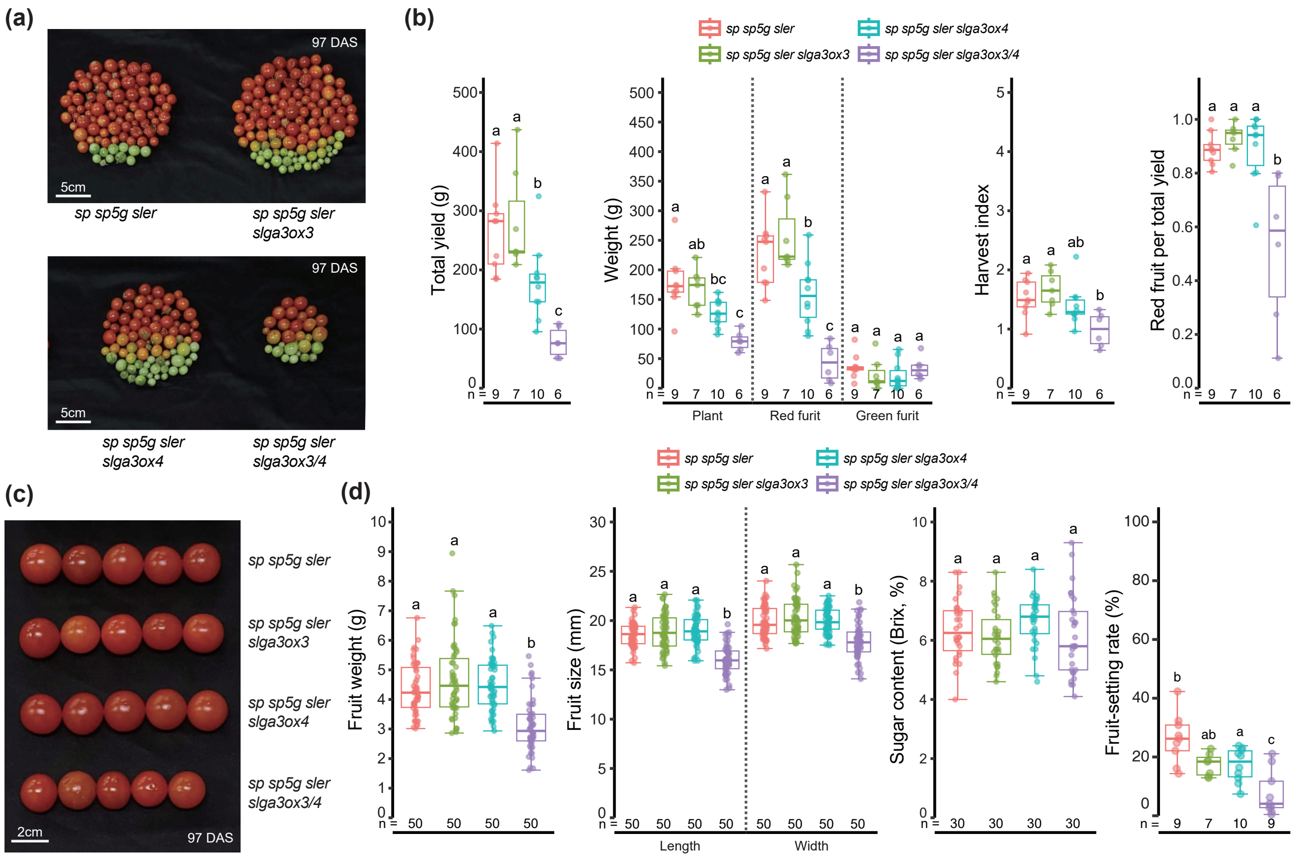

Figure S9. Yield potential analysis of *slga3ox3, slga3ox4,* and *slga3ox3/4* mutants. (a) Total fruits of *sp sp5g sler, sp sp5g sler slga3ox3, sp sp5g sler slga3ox4,* and *sp sp5g sler slga3ox3/4*. (b) Total yield, weight, harvest index, and red fruits per total yield of *sp sp5g sler, sp sp5g sler slga3ox3, sp sp5g sler slga3ox4,* and *sp sp5g sler slga3ox3/4*. (c) The five fruits with the average width of *sp sp5g sler, sp sp5g sler slga3ox3, sp sp5g sler slga3ox4*, and *sp sp5g sler slga3ox3/4*. (d) Fruit weight, fruit length, sugar content, and fruit-setting rate of *sp sp5g sler, sp sp5g sler slga3ox3, sp sp5g sler slga3ox4*, and *sp sp5g sler slga3ox3/4*. (a–d) The plants grown from May 09, 2023 to August 14, 2023. (b–d) Box plots, 25th–75th percentile; center line, median; whiskers, full data range. Exact sample sizes (n) for replicate types are indicated. Letters indicate significance groups at $P < 0.05$ (one-way ANOVA and Tukey HSD). Different letters between genotypes indicate significance. At least twice experiments were repeated independently with similar results.
